# Radiodynamic Therapy Using TAT Peptide-Targeted Verteporfin-Encapsulated PLGA Nanoparticles

**DOI:** 10.3390/ijms22126425

**Published:** 2021-06-15

**Authors:** Sandhya Clement, Ayad G. Anwer, Layla Pires, Jared Campbell, Brian C. Wilson, Ewa M. Goldys

**Affiliations:** 1 ARC Centre of Excellence in Nanoscale Biophotonics, The Graduate School of Biomedical Engineering, University of New South Wales, Sydney, NSW 2052, Australia; a.anwer@unsw.edu.au (A.G.A.); j.campbell@unsw.edu.au (J.C.); e.goldys@unsw.edu.au (E.M.G.); 2 ARC Centre of Excellence in Nanoscale Biophotonics, Department of Physics and Astronomy, Macquarie University, Sydney, NSW 2109, Australia; 3 Princess Margaret Cancer Centre, University Health Network and Department of Medical Biophysics, University of Toronto, Toronto, ON M5S 1A1, Canada; Layla.Pires@uhnresearch.ca (L.P.); Brian.Wilson@uhnresearch.ca (B.C.W.)

**Keywords:** reactive oxygen species, ROS, singlet oxygen, nanoparticles, radiation, RDT, radiosensitization, photosensitizer, PLGA, X-PDT, nuclear targeting, TAT peptide, verteporfin, radiation therapy, radiodynamic therapy

## Abstract

Radiodynamic therapy (RDT) is a recent extension of conventional photodynamic therapy, in which visible/near infrared light irradiation is replaced by a well-tolerated dose of high-energy X-rays. This enables greater tissue penetration to allow non-invasive treatment of large, deep-seated tumors. We report here the design and testing of a drug delivery system for RDT that is intended to enhance intra- or peri-nuclear localization of the photosensitizer, leading to DNA damage and resulting clonogenic cell kill. This comprises a photosensitizer (Verteporfin, VP) incorporated into poly (lactic-co-glycolic acid) nanoparticles (PLGA NPs) that are surface-functionalized with a cell-penetrating HIV trans-activator of transcription (TAT) peptide. In addition to a series of physical and photophysical characterization studies, cytotoxicity tests in pancreatic (PANC-1) cancer cells in vitro under 4 Gy X-ray exposure from a clinical 6 MV linear accelerator (LINAC) showed that TAT targeting of the nanoparticles markedly enhances the effectiveness of RDT treatment, particularly when assessed by a clonogenic, i.e., DNA damage-mediated, cell kill.

## 1. Introduction

Photodynamic therapy (PDT) is an approved modality for several pre-cancerous lesions and solid tumors [1,2], and has non-oncological applications [3]. It employs a photosensitizer (PS) activated by visible or near-infrared light, most commonly to generate a singlet oxygen (^1^O_2_), a highly cytotoxic reactive oxygen species (ROS) [4]. However, PDT has had limited clinical application for treating deep-seated, large or disseminated tumors, due to the shallow penetration of light in tissue that requires technically challenging endoscopic or interstitial fibre-optic light delivery. The strong tissue- and wavelength-dependence of the optical absorption and scattering also make it difficult to achieve complete tumor ablation [5]. In addition, the hydrophobic nature of many molecular PSs leads to aggregation in biological media, requiring formulations such as liposomes for efficient delivery to tumors in vivo in order to achieve the maximum treatment efficacy [6].

Radiodynamic therapy (RDT), sometimes also referred to as X-PDT, is a modification of conventional PDT in that the external activating light source is replaced by high-energy X-rays, such as those produced by a linear accelerator (LINAC) used in radiotherapy or by suitable radioisotopes. This concept was introduced by Chen et al. in 2006 [7] and has since been investigated by a number of groups. It has showed a range of efficacies in vitro or in vivo, as reviewed in [8,9,10], including the use of nanoparticle formulations of the photosensitizer [11,12,13,14,15]. We previously reported that the photosensitizer Verteporfin, which is already used for liposomal formulation in clinical practice, can function as an RDT agent, which is important for accelerating the eventual clinical translation of this modality [16,17,18,19]. Similar findings for other PSs (e.g., protoporpyrin IX) have been reported by other groups [20,21,22]. As we have recently reviewed in detail [23], the mechanisms of ROS generation in RDT are not fully understood but include direct PS activation by secondary electrons, the use of scintillation nanocrystals to convert X-ray energy into light, and PS activation by the Cerenkov light generated by high-energy secondary electrons produced in tissue by MeV X-rays.

Targeted PS delivery systems using antibodies or peptides have been studied in PDT [24,25] as well as in RDT to increase uptake and selectivity [8,26]. Of particular interest here, among different cell-penetrating peptides (CPP), the TAT (transactivator of transcription) peptide is known to be an efficient targeting moiety for translocating nanoparticles into the cell nucleus via binding to importin α and β (karyopherin) receptors [27,28,29,30,31,32]. Nuclear targeting in the context of conventional, light-activated PDT has been described in the earlier literature [33,34,35,36], where markedly increased photocytotoxicity due to direct DNA damage was reported, with up to a >1000-fold reduction in PS dose to achieve the same in vitro cell kill as that of a non-targeted PS. This approach had not been reported in the context of RDT, which is the focus of this paper.

For this purpose, we have developed a polymer nanoparticle-based PS formulation for delivery to the tumor, with a TAT peptide as a nuclear localizing signal. It is based on polylactic-co-glycolic acid (PLGA), an FDA-approved elastomeric copolymer specifically used for drug delivery owing to its biocompatibility and biodegradability. PLGA was used here to encapsulate Verteporfin (VP) [37], and it is expected that these PLGA nanoparticles (NPs) will allow the encapsulated drugs to accumulate in the tumor in vivo through the well-known enhanced permeability and retention (EPR) effect [38,39]. They could also be conjugated into tumor cell-targeting moieties to increase further their efficacy and specificity. Here, a series of in solu characterization studies were performed, including NP sizing and zeta potential and stability in biological media. The cellular uptake and intracellular localization of the PLGA–VP–TAT nanoparticles were then measured as well as the resulting ^1^O_2_ generation and in vitro RDT responses in human pancreatic cancer (PANC-1) cells. The RTD cytotoxicity was measured by several assays: lipid peroxidation, MTS, apoptosis/necrosis and, most importantly, DNA double-strand breaks and clonogenicity. These initial studies reported here focus on comparing the RDT efficacy of PLGA–VP–TAT) vs. PLGA–VP.

## 2. Results

### 2.1. PLGA–VP and PLGA–VP–TAT Nanoparticle Characterisation

**Figure 1:**
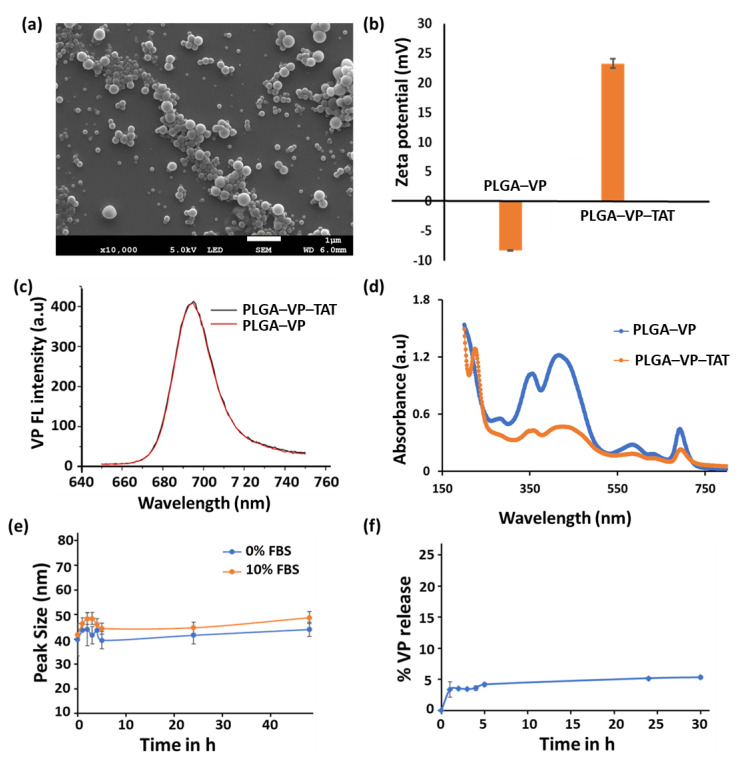
Characterization of PLGA–VP–TAT and PGLA–VP nanoparticles in solution (**a**) SEM image, (**b**) Zeta potential in water, (**c**) florescence spectra of VP (425 nm ex/690 nm em) from the NPs in water, (**d**) absorbance spectra in water, showing the VP and TAT peaks, (**e**) PLGA–VP–TAT NP stability in PBS with and without FBS, as measured by the nanoparticle peak size, (**f**) % release of VP from PLGA–VP–TAT NPs in PBS at 37 °C.

An SEM image of the PLGA–VP NPs is shown in Figure 1a. The nanoparticles were mostly spherical with an average diameter of 85 ± 12 nm. TAT conjugation changed the value of the zeta potential from (−8.23 ± 0.05) mV to (+2.3 ± 0.8) mV (Figure 1b), which was attributable mainly to the positively charged TAT amino acids [40]. VP fluorescence (425 nm excitation, 690 nm emission) was seen with both PLGA–VP and PLGA–VP–TAT NPs (Figure 1c) and allowed imaging of the cell uptake and localization [16]. The presence of the TAT peptide on the surface of the PLGA–VP–TAT NPs was confirmed by absorption spectroscopy (Figure 1d and Supplementary Figure S1) and FTIR spectroscopy (Supplementary Figure S2). The peak size of the PLGA–VP–TAT NPs in PBS with and without 10% FBS showed a slight increase during the first 4 h but then remained constant for the next few 48 h (Figure 1e), indicating that there was no significant aggregation in biological media. The % release of VP from the PLGA-VP NPs in PBS suspension at 37 °C was plotted as a function of time (Figure 1f), and showed that, after some initial release in the first 4 h, >95% of the VP remained trapped within the NPs (Figure 1e), likely due to VP being highly hydrophobic with a low affinity for the hydrophilic environment outside the PLGA matrix [41]. This drug release profile is consistent with our previous findings [17].

### 2.2. Cellular Uptake

**Figure 2:**
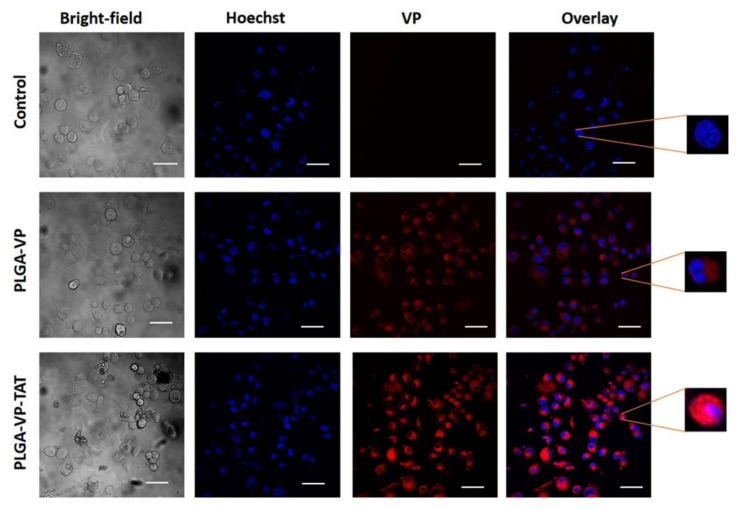
Representative confocal images showing the uptake of PLGA–VP and PLGA–VP–TAT nanoparticles in PANC-1 cells after 4 h incubation: blue-Hoechst nuclear stain, red-VP fluorescence. Magnified single-cell images are shown on the right. Scale bar: 50 µm.

The uptake of the PLGA–VP and PLGA–VP–TAT NPs was measured at 4 h incubation, which was found previously to be optimal (i.e., gave the maximum NP uptake) [17]. As seen in Figure 2, there was no measurable VP signal present in the control group (cells only). The signal was ~2-fold higher for PLGA–VP–TAT NPs than for the untargeted NPs (*p* = 0.009) (Supplementary Figure S3) and the magnified single-cell images with TAT targeting showed some VP signal within the nuclei. The enhanced cellular uptake of the TAT-targeted NPs is attributed to the stronger adsorption onto the cell membrane due to electrostatic interaction between the cationic TAT peptide and heparan sulfate proteoglycans (HSPGs) on the cell surface [42].

### 2.3. RDT Singlet Oxygen Generation and In Vitro Cytotoxicity

**Figure 3:**
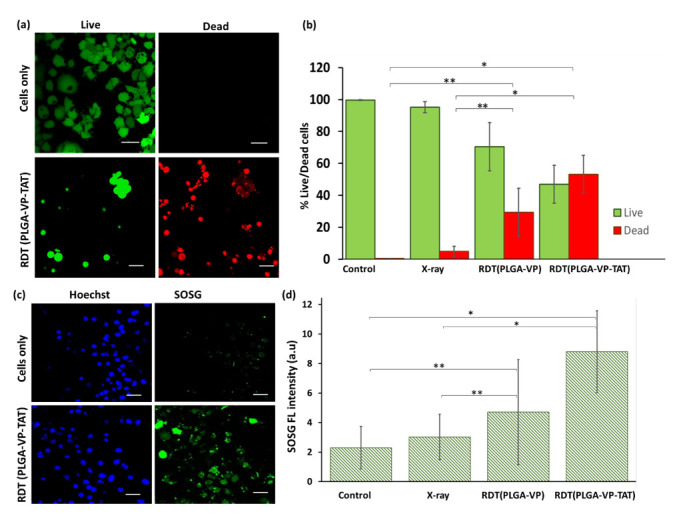
Cell viability and singlet oxygen generation following RDT. (**a**) representative confocal fluorescence image of live (green) and dead (red) cells following RDT with PLGA–VP–TAT NP and 4 Gy, compared to no-treatment controls, (**b**) corresponding cell viability, (**c**) representative confocal fluorescence image of SOSG fluorescence (green) and nuclear staining (blue) following 4 Gy RDT with PLGA–VP–TAT NPs compared to untreated controls, (**d**) SOSG fluorescence in treated and control cells, * *p* < 0.05 and ** *p* < 0.5. Scale bar: 50 µm.

The in vitro therapeutic efficacy of RDT was assessed. The NP dark toxicity and cell viability after RDT were measured first using the MTS assay. This showed (Supplementary Figure S4) that, in the absence of X-ray treatment, the nanoformulation resulted in cell viability of ~90% for PLGA–VP–TAT NPs and >95% for PLGA–VP NPs and PLGA NPs without VP. For RDT (at 4 Gy) the cell viability was reduced to 75 ± 8% (*p* = 0.02) and 62 ± 5% (*p* = 0.007) with PLGA-VP NPs and PLGA–VP–TAT NPs, respectively. The results of the live/dead cell viability assay are shown in Figure 3a, where it is evident that most of the control (untreated) cells were viable, whereas >50% of the cells were dead following RDT with PLGA–VP–TAT NPs (*p* = 0.02). Detailed images, including all different control groups, are provided in the Supplementary Figure S5. The results for the various treatment and control groups are shown in Figure 3b. In particular, the cell death for RDT treatments with PLGA–VP–TAT or PLGA-VP NPs were 53 ± 12% and 29 ± 15%, respectively.

These results are consistent with the singlet oxygen generation in the cells, as monitored with the fluorescent SOSG probe: Figure 3c,d. Supplementary Figure S6 shows the confocal images of SOSG fluorescence of the various control groups. Less than 5% increase in SOSG signal was seen with X-rays only compared to untreated control cells, and increased by ~105% and ~280% in cells undergoing RDT with PLGA–VP NPs and PLGA–VP–TAT NPs, respectively.

### 2.4. DNA and Lipid Membrane Damage

**Figure 4:**
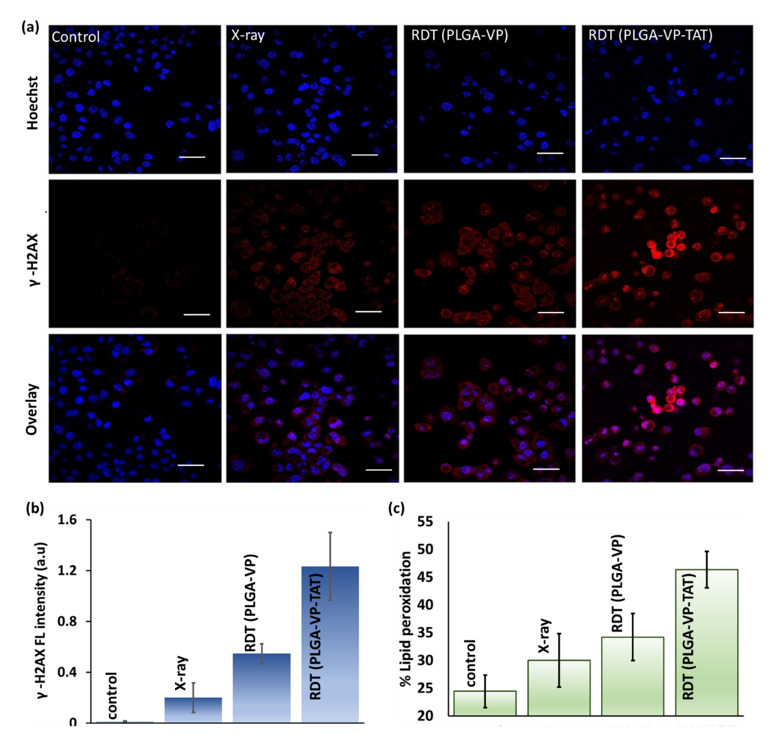
RDT effects at the cellular level. (**a**) representative confocal microscopy images showing DNA double strand breaks indicated by γ-H2AX staining (red: γ-H2AX, blue: Hoechst nuclear stain), (**b**) quantification of γ-H2AX fluorescence, (**c**) lipid peroxidation in the treatment and control groups. Scale bar: 50 µm.

Double-strand breaks (DSBs), the most common lethal lesion in DNA following ionizing radiation exposure [43], were measured following RDT by detecting the presence of γ-H2AX, a phosphorylated form of the histone H2A variant that forms rapidly at the sites of DNA DSBs [44]. The results (Figure 4a,b) indicate minimal DNA damage in untreated control cells. RDT with PLGA–VP NPs resulted in ~2.5-fold increase in signal compared with radiation (4 Gy) alone (*p* = 0.01), while RDT using the PLGA–VP–TAT NPs showed ~6-fold increase (*p* = 0.006 compared to radiation alone; *p* = 0.04 compared to RDT with untargeted NPs).

RDT-induced apoptosis and necrosis are shown in Supplementary Figure S7: cells treated with nuclear-targeted RDT showed significant higher levels of necrotic cell death than non-targeted RDT or controls, with necrosis being more pronounced than apoptosis. This reduction in the cell membrane integrity is supported by the measurement of lipid peroxidation [45] shown in Figure 3d and discussed more in Supplementary Section S8 (Figure S8). Figure 4b shows ~90% increase in lipid peroxidation for RDT using the TAT-targeted NPs compared to untreated controls, which was significantly higher (*p* < 0.05) than RDT with the untargeted PLGA–VP NPs (~40%) or with radiation alone (~20%).

### 2.5. Clonogenic Response to RDT

**Figure 5:**
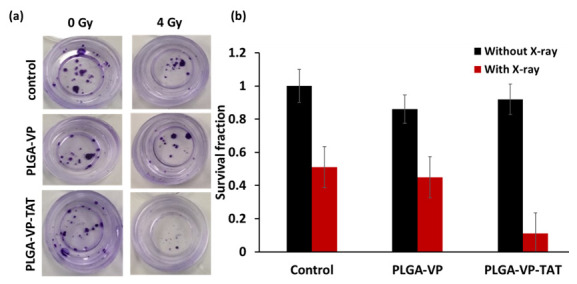
Clonogenic assay. (**a**) example of stained colonies at 14 d following treatment, (**b**) survival fraction for RDT in various treatment groups.

The cell viability assays above do not determine the clinically important long-term response of cancer cells after treatment [12] since they do not account for altered proliferative capacity or repair of sublethal damage [46]. The results of the clonogenic assay [47] are shown in Figure 5. There was a significant difference in the number of colonies (colonies > 50 cells) following RDT treatment with PLGA–VP–TAT NPs compared to RDT with untargeted NPs (*p* < 0.005), X-rays only (*p* < 0.001), and no treatment (*p* < 0.001).

## 3. Discussion and Conclusions

The PLGA-based VP nanoformulation (PLGA–VP) with spherical nanoparticles <~100 nm diameter was synthesized by an emulsion–evaporation technique. The various characterization metrics (absorption, fluorescence, FTIR) showed that the hydrophobic VP molecules were encapsulated successfully within the nanoparticles. Conjugation of the TAT peptide on the nanoparticle surface resulted in a stable PLGA–VP–TAT NP formulation as confirmed by the Zeta potential and absorption measurements over time. The uptake of the nanoparticles in human PANC-1 pancreatic cancer cells was significantly higher with TAT targeting, including within the cell nuclei, compared to the non-targeted VP nanoformulation and resulted in significantly greater RDT cytotoxicity as reflected in both higher DNA and cell membrane damage. With targeting, RDT was markedly more cytotoxic than the same dose of X-rays without nanoparticles, which was attributed to increased radiation-induced ROS generation, specifically singlet-oxygen generation from the VP molecules.

Most importantly for potential clinical relevance, the clonogenic assay results showed that the cells had only about 10% proliferative capacity after a single 4 Gy dose of TAT–NP-targeted RDT. This is a substantially higher efficacy than the cytotoxicity measured by the other cell-death assays that depend on damage to extra-nuclear organelles. In interpreting this result it should be noted that, consistent with the reported size-dependent nuclear uptake of nanodrugs facilitated by the TAT peptide [48], not all the targeted NPs were localized in the nucleus or perinuclear region; hence, the current formulation was not fully optimized for nuclear-targeted RDT. Reducing the nanoparticle size may increase the cellular and nuclear uptake and enhance the RDT efficacy further, and these investigations are in progress. On the other hand, it may be desirable to have some degree of non-nuclear cell-death pathways (in particular, necrosis) to retain the advantage of immune modulation, as seen in conventional PDT, to contribute to anti-tumor efficacy [49,50,51,52].

In comparing these results with other studies of RDT, those using psoralens are of note [53]. Psoralens are used clinically in PUVA (Psoralen-UVA) phototherapy for a variety of benign skin conditions. They work by intercalating into DNA so that cell proliferation is blocked under ultraviolet-A exposure. X-ray activation of a psoralen, mediated through MeV X-ray-generated Cherenkov light, showed increased cell death in vitro (as measured by an adenosine triphosphate luminescence assay of around 20% in breast cancer and 9.5% in melanoma cells) using 50 µM psoralen and a 2 Gy X-ray dose. Interestingly, also seen was a 4- to 8-fold increase in the surface expression of major histocompatibility complex I (MHC I), which is related to immune response [53]. In vivo studies using a syngeneic 4T1 breast cancer model showed that a psoralen-based RDT reduced tumor growth [54].

In general, RDT in its various forms with different photosensitizers has shown both in vitro and in vivo efficacy across in a range of tumor types [16,17,18,20,21,22,55,56,57,58]. Nevertheless, it is not clear that the efficacy is enough to translate into a stand-alone treatment using a well-tolerated (e.g., <10 Gy) dose of high-energy (MeV) X-rays combined with systemic administration of an approved clinical photosensitizer. There is, however, still the option to use phototherapeutic agents as a novel form of radiation sensitizer for use in conventional fractionated radiation therapy depending on how often the photosensitizer needs to be administered. The findings from our initial study here suggest that active targeting of nanoparticles incorporating the photosensitizer could be an effective option to enhance the efficacy of RDT, especially to destroy the proliferative capacity of tumor cells.

We recognize that the above results, while encouraging, do not represent definite proof of nuclear-targeted RDT, since there are several potentially confounding effects that need to be considered. Thus, while the fluorescence microscopy of the VP distribution suggests a higher nuclear localization with the TAT-conjugated NPs, true quantification in this approach is challenging [59]. Cellular disaggregation followed by quantitative fluorimetry of the organelle fractions [60] would be a more robust assay. In addition, the fact that the DNA double-strand breaks increased compared to the sole X-ray control, even without the TAT, suggests that other effects may be occurring. For example, X-irradiation is known to increase cellular and nuclear membrane permeability [61,62] such that it could allow a degree of nuclear internalization even without the TAT. The greater DNA damage with the TAT NPS may then reflect simply a greater total-cell uptake rather than a specific nuclear localization. One way to control this would be to measure accurately the total cell uptake of the NPs and then adjust the relative concentration of the targeted and untargeted NPs to give the same total uptake at the time of X-irradiation. Again, such experiments are planned.

The next steps also include further optimization of the nanoformulation and evaluation of RDT efficacy in vivo and to other tumor types. If successful, a nuclear-targeted RDT has potential as a novel treatment option, either as a stand-alone or to enhance the efficacy of fractionated radiotherapy, including in the treatment of deep-seated tumors such as in the pancreas.

Finally, as with most anti-cancer therapies, the tumor selectivity of nuclear-targeted radiodynamic therapy will be an important issue for clinical translation. Even with accurate spatial localization of current radiation therapy delivery, if there is significant nanoparticle accumulation in normal organs, there will be activation and potential cytotoxicity in normal tissue lying along the radiation beam paths in addition to that caused by the radiation, itself. Hence, it is likely that tumor-cell targeting, in addition to nuclear localization by the TAT or other nuclear-localizing strategies, will be required to achieve differential uptake beyond that caused by the EPR effect. There is also the risk of enhanced skin phototoxicity due to nuclear uptake that must be addressed. Direct intratumoral administration of nanoparticles or intra-arterial delivery may be options to mitigate these off-target toxicities. The dark (no light, no X-rays) toxicity should, on the other hand, be minimal, given the excellent safety profile of the components in the formulation

## 4. Materials and Methods

### 4.1. Materials

Resomer^®^ RG 504 H; Poly(D,L-lactide-co-glycolide) (PLGA; #719900), poly(vinyl alcohol) (PVA; #363138), dichloromethane (DCM; #650463), Verteporfin (VP; #SML0534), dimethyl sulfoxide (DMSO; #D2650), 1-[3-(Dimethylamino)propyl]-3-ethylcarbodiimide methiodide (EDC; #165344), N-Hydroxysuccinimide (NHS; #130672), human pancreatic ductal adenocarcinoma cell line (PANC-1), Dulbecco’s Modified Eagle’s Medium—high glucose without L-Glutamine (#D5671) and Gentian violet (#G2039) were purchased from Sigma-Aldrich (St. Louis, MI, USA). Singlet oxygen sensor green probe (SOSG; #S-36002), NucBlue™ Live ReadyProbes™ Reagent (#R37605), LIVE/DEAD™ Viability/Cytotoxicity Kit for mammalian cells (#L3224), HCS DNA Damage Kit (H10292), Image-iT™ Lipid Peroxidation Kit for live cell analysis (C10445), DPBS with calcium and magnesium (#14040182), DPBS with no calcium or magnesium (#14190250), TrypLE™ Express Enzyme (1X) with no phenol red (#12604021), L-Glutamine, 200 mM (#25030081) and Fetal Bovine Serum (FBS) qualified US origin (#26140079) were purchased from Life Technologies (Carlsbad, CA, USA). Apoptosis/Necrosis Assay Kits (blue, red, green) (ab176750) and CellTiter 96^®^ AQueous One Solution Assay were purchased from Abcam (Cambridge, UK) and Promega (Madison, WI, USA) respectively. Except as otherwise noted, all commercial assays were performed according to the manufacturers’ instructions.

### 4.2. Methods

#### 4.2.1. Nano Formulation and Conjugation

We prepared PLGA, PLGA-VP and PLGA–VP–TAT nanoparticles using a published solvent-evaporation single-emulsion method with slight modifications [17]. Briefly, a 3 mM stock solution of VP (2.2 mg/1 mL) in DMSO and 5% PVA (5 g/100 mL) solution in water were prepared. Later, 30 mg of PLGA was dissolved in 3 mL of DCM and 200 µL of VP stock was added dropwise while vortexing. This mixture was added dropwise to 12.5 mL of 5% PVA and vortexed thoroughly before sonication for 1.5 min (3 × 30 s) at an output power of 200 W using a microtip-probe sonicator (Branson Digital Sonifier, S-250D; Emerson Industrial Automation, CT, USA). The resulting solution was placed overnight in a shaker (300 rpm) to evaporate the DCM, after which it was centrifuged at 12,500 rpm for 20 min and redispersed in water. This washing procedure was repeated twice and finally the NPs were redispersed in 12 mL water to form the PLGA–VP NPs and stored in the dark at 4 °C. A PLGA NP stock solution was also prepared using the same procedures except for the addition of VP and served as a control.

**Figure 6:**
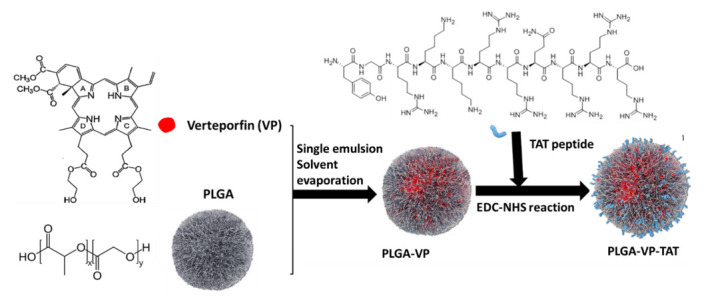
Schematic illustration of PLGA–VP–TAT nanoparticle formulation.

The PLGA–VP–TAT nanoparticles were prepared by conjugating the TAT peptide to the nanoparticle surface through an EDC–NHS reaction. EDC couples NHS to a carboxylic group in the PLGA, forming a highly stable NHS ester, and this allows efficient conjugation to primary amines in the TAT peptide. The conjugation procedure was as follows: 2 mL of PLGA–VP stock was centrifuged, the supernatant was removed, and 1.2 mL PBS was added. Then, 30 mg of NHS and 60 mg of EDC were added before incubation for 2 h in an orbital shaker (200 rpm). A 100 µM TAT stock was first prepared, then 60 µL of TAT stock in 200 µL of PBS was added. This mixture was kept overnight in a shaker (150 rpm), washed, centrifuged at 12,500 rpm for 10 min, redispersed in 2 mL of DI water and stored in the dark at 4 °C. The main formulation steps are illustrated in Figure 6.

#### 4.2.2. Characterization

The size and zeta potential of the NPs were measured using dynamic light scattering (DLS) (Zetasizer Nanoseries; Malvern Panalytical Ltd, Malvern, Worcestershire, UK). All measurements were performed in triplicate at 25 °C. The fluorescence emission spectrum of VP (425 nm excitation) in various nanoformulations was measured in a spectrofluorometer (Cary Eclipse Spectrophotometer; Varian, Inc., Palo Alto, CA, USA) in a quartz cuvette at room temperature. The absorption spectra of different NPs were measured in a double-beam spectrophotometer (Cary UV–VIS–NIR; Agilent, Santa Clara, CA, USA) using 1 cm pathlength quartz cuvettes. The conjugation of TAT to the surface of the NPs was confirmed using both absorption and Fourier Transform InfraRed (FTIR) spectroscopy.

#### 4.2.3. In vitro VP Release from PLGA NPs

To begin, 500 μL of PLGA–VP–TAT NPs was centrifuged and redispersed in PBS, with and without 10% FBS, to mimic physiological conditions. The solution was placed in a Lyzer tube (Midi Pur-A-Lyzer 6000 Dialysis Kit) that was placed in a 50 mL tube with 15 mL of PBS and incubated at 37 °C for different times (1, 2, 3, 4, 5, 24 and 30 h). An aliquot of PBS was taken for fluorescence measurement of the released VP at each time point. The percentage of VP release was calculated as

$$\%\;VP\;release=\frac{100\;\left(VP_{F}\right)}{\left(VP_{c}\right)}$$

where $VP_{F}$ and $VP_{c}$ represent, respectively, the peak fluorescence emission intensities of VP in PBS and in a control sample that was prepared by dispersing PLGA–VP NPs in 15 mL PBS and 200 μL DMSO.

#### 4.2.4. Nanoparticle Stability

The stability of the PLGA–VP–TAT NPs in PBS, with and without 10% FBS, was measured as follows. 1 mL PLGA–VP–TAT in water was centrifuged and then dispersed in the media and then kept in a water bath at 37 °C, with the size was checked at regular intervals up to 48 h to test for aggregation.

#### 4.2.5. X-Irradiation

All irradiations were performed using a clinical 6 MV LINAC at the Genesis Cancer Care, Macquarie Hospital, Sydney, Australia at 4 Gy, representing a well-tolerated low dose compared to a typical clinical dose of 50–70 Gy in 2 Gy fractions over several weeks.

#### 4.2.6. Cell Culture

Human pancreatic cancer cells, PANC-1 were grown at 37 °C with 5% CO_2_ in media comprising DMEM (without L-glutathione) mixed with 10% FBS, 1% antibiotic–antimycotic and 1% L-glutathione. At 80% confluence, the cells were transferred either to 35 mm petri dishes or 96-well plates, depending on the experiment. A 4 µM concentration of VP was used for all in vitro studies and all experiments were performed under minimum lighting conditions to minimize photobleaching and direct PDT effects. Laser-scanning confocal fluorescence microscopy (FV3000; Olympus, Tokyo, Japan) was used in various stages of the PS uptake and in vitro RDT-response assays, with appropriate excitation and detection wavelengths, for which the microscope settings (laser power, photodetector gain, integration time) were fixed for each assay. Image J software was used for analysis and quantification.

#### 4.2.7. Singlet Oxygen Measurements

The singlet oxygen sensor green probe (SOSG) was used to detect ^1^O_2_ generation in solu and in vitro, measuring its fluorescence emission at 525 nm under 488 nm excitation. For this, a stock solution of SOSG (500 µM) was made by adding 660 µL methanol into an SOSG vial and diluting it to 8 µM that was found previously to be optimal both in solu and in vitro [15]. Controls included NPs only, X-rays only and cells only.

#### 4.2.8. Nanoparticle Cellular Uptake and Localization

Cells were seeded into 35 mm^2^ petri dishes and grown to 60% confluence. The growth medium was replaced with a fresh medium containing the NPs and the cells were incubated for 4 h. The control cells were kept in a fresh culture medium without NPs. Cells were then washed 3 times. Fresh medium was added, and the cells were incubated overnight. NucBlue™ Live ReadyProbes™ Reagent was added to stain the cell nuclei and the cells were imaged with 405 nm excitation, measuring the fluorescence (430–480 nm) in the nuclear region as well as the VP fluorescence (650–700 nm).

#### 4.2.9. Viability MTS Assay

Cells were seeded in 96-well plates at 10,000 cells/well and incubated for 4 h with or without NPs. The treatment (with PLGA–VP–TAT NPs) and controls (cells only, cells with PLGA NPs, cells with PLGA–VP NPs) were X-irradiated. The cell viability was measured immediately using the MTS assay (CellTiter 96^®^ AQueous One Solution), reading the absorbance at 490 nm on a plate reader. Cell viability was calculated relative to non-treatment controls.

#### 4.2.10. Live/Dead Assay

A LIVE/DEAD™ Viability/Cytotoxicity Kit was used to assess the RDT cytotoxicity. Cells were seeded at 1 × 10^5^ cells/mL in a 35 mm petri dish and grown to 60% confluence. After NP incubation for 4 h followed by X-irradiation, the assay was run (following the manufacturer’s protocol), and the cells were imaged (488 nm excitation and 520 and 625 nm emission).

#### 4.2.11. Apoptosis/Necrotic Assay

Using the same cell preparation and treatments as for the live/dead assay, the apoptosis/necrotic assay (ab176750) was performed immediately after X-irradiation. The cells were imaged and live (405 nm excitation/450 nm emission), apoptotic (490 nm/525 nm) and necrotic (630 nm/690 nm) cells were counted.

#### 4.2.12. DNA Damage Assay

Double-strand DNA breaks were assayed using the HCS DNA damage kit (H10292) immediately following RDT treatment, by immunostaining (according to the manufacturer’s protocol) for phosphorylated γH2AX and imaging the cell fluorescence (555 nm excitation, 565 nm emission).

#### 4.2.13. Lipid Peroxidation Assay

The Image-iT™ Lipid Peroxidation Kit for live cell analysis was used to assess the peroxidation of lipid membranes. In this assay, the fluorescence from live cells shifted from red (590 nm) to green (510 nm) under excitation at 580 and 488 nm, respectively. The cells were prepared as for the live/dead cell assay, and the red/green, i.e., reduced/oxidized, ratiometric image yielded the percent lipid peroxidation relative to untreated controls.

#### 4.2.14. Long-Term Proliferation

Clonogenicity was used to assess the long-term proliferative capacity of the cells. Following RDT treatment with a starting density of 500 cells/dish, cells were incubated for 2 weeks, with the medium changed every other day. After day 14, the cells were fixed with 4% formaldehyde at room temperature for 15 min, followed by washing with PBS (+Ca and +Mg). Colonies were then stained with Gentian violet (5 drops per dish) for 30 min, washed to remove excess stain and dried at room temperature for 1 h. Colonies with >50 cells were then counted manually and the survival fraction was calculated as [63]

$$\mathrm{Survival}\;\mathrm{fraction}=\frac{\mathrm{Number}\;\mathrm{of}\;\mathrm{colonies}\;\mathrm{formed}\;\mathrm{after}\;\mathrm{treatment}}{\mathrm{No}\;\mathrm{of}\;\mathrm{cells}\;\mathrm{seeded}\times \mathrm{Plating}\;\mathrm{efficiency}}$$

where the plating efficiency is the percentage of seeded cells that survived to form colonies under no-treatment conditions.

#### 4.2.15. Statistical Analysis

All measurements were repeated at least in triplicate, and results were presented as a mean ± 1 standard deviation. Two sample *t*-tests were applied, with *p* < 0.05 being considered statistically significant.

## Data Availability

The data presented in this study are available within the article.

## Supplementary Materials

The following are available online at https://www.mdpi.com/article/10.3390/ijms22126425/s1.
