# Supplementary material for: Radiodynamic Therapy Using TAT Peptide-Targeted Verteporfin-Encapsulated PLGA Nanoparticles
Source: Int J Mol Sci. 2021 Jun 15;22(12):6425. doi: 10.3390/ijms22126425 (PMC8232618; doi:10.3390/ijms22126425)
Supplement: Supplementary file 1 [file ijms-22-06425-s001.zip › ijms-1243311-supplementary.pdf]

RadioDynamic Therapy using TAT Peptide-Targeted Verteporfin- Encapsulated PLGA Nanoparticles.

Sandhya Clement[1,2], Ayad Anwer[1,2], Layla Pires[3], Jared Campbell[1,2], Brian C Wilson[3] and Ewa M. Goldys[1,2]

[1] ARC Centre of Excellence in Nanoscale Biophotonics, The Graduate school of Biomedical Engineering, University of New South Wales, Sydney, Australia

[2] ARC Centre of Excellence in Nanoscale Biophotonics, Department of Physics and Astronomy, Macquarie University, Sydney, Australia

[3]Princess Margaret Cancer Centre, University Health Network and Department of Medical Biophysics, University of Toronto, Ontario, Canada

Supplementary material

S1. Absorption Spectra of PLGA-VP-TAT NPs:

Fig. S1 shows the optical absorption peaks of VP and TAT [1] in the PLGA-VP-TAT nanoparticles, indicating that TAT was successfully conjugated to the nanoparticle surface.

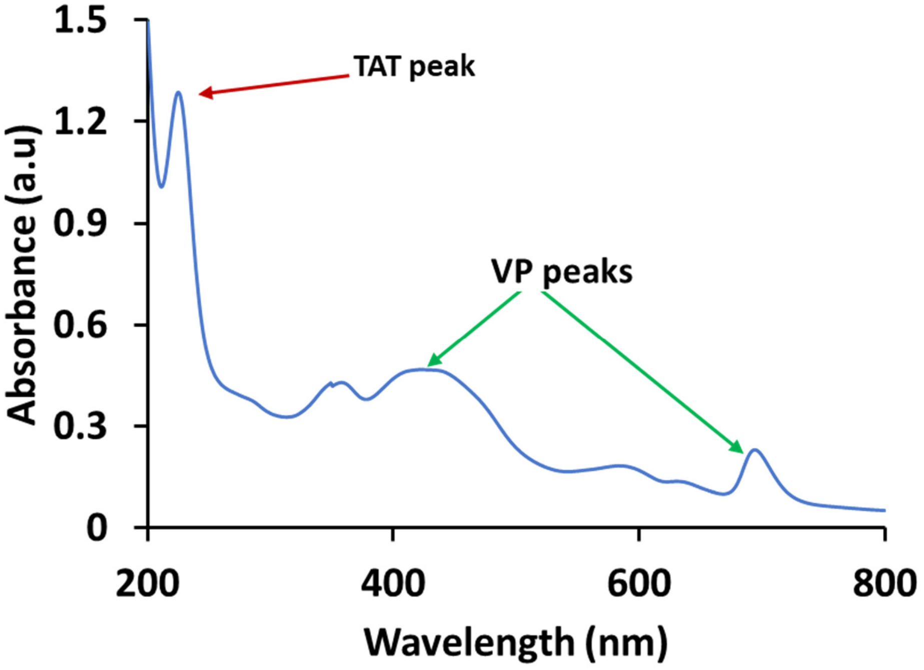

Figure S1*: Optical absorption spectra of PLGA-VP-TAT indicating the VP and TT peptide peaks.*

S2. FTIR Spectra:

The FTIR spectra in Fig. S2 show the peaks of PLGA, VP, PLGA-VP, TAT and PLGA-VP-TAT nanoparticles.

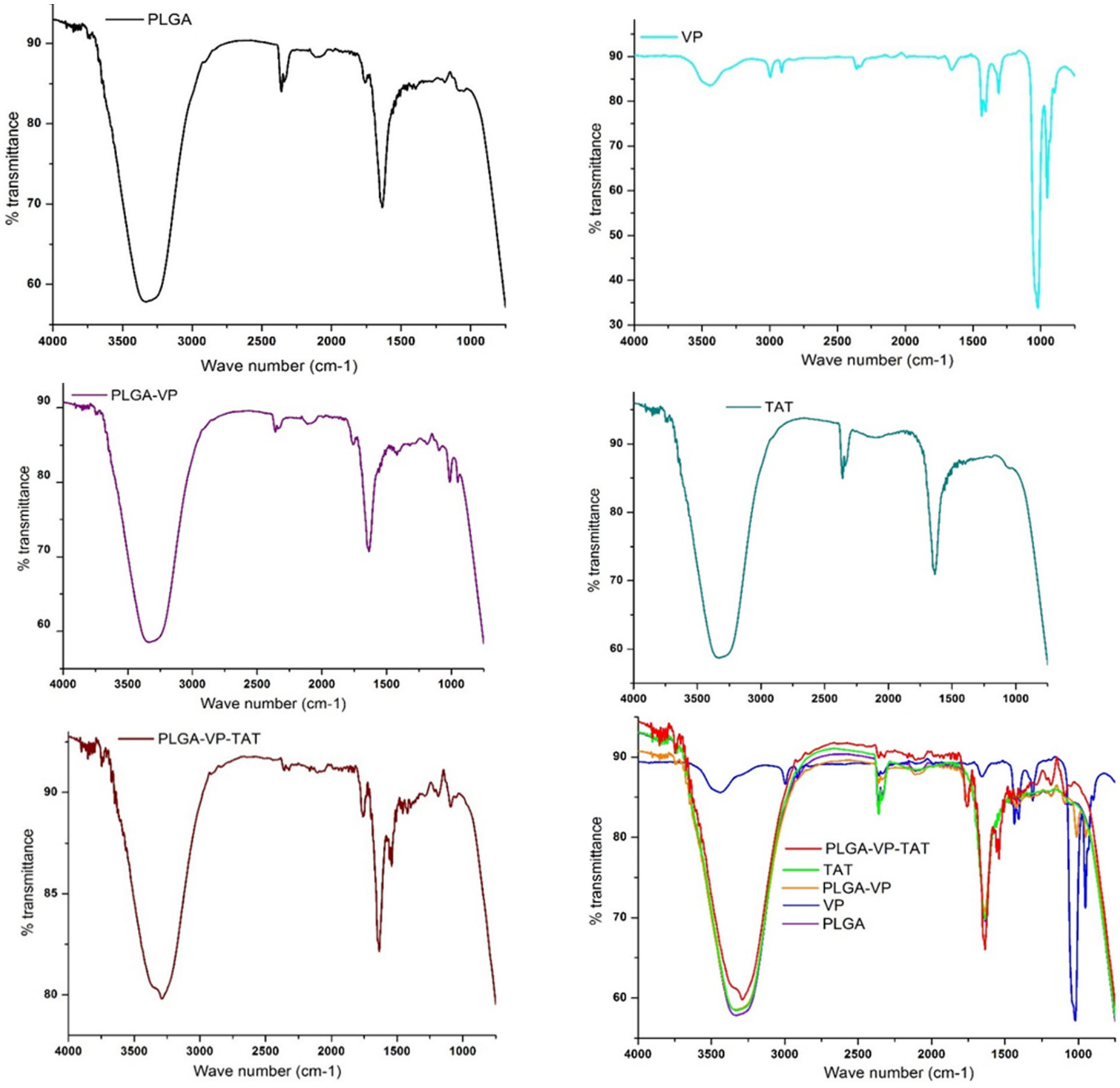

Figure S2. *FTIR spectra of PLGA, VP, PLGA-VP, TAT, PLGA-VP-TAT and their combined spectra.*

S3. Cell uptake:

Amount of verteporfin inside the PANC-1 cells were assessed from the confocal microscopy images taken as shown in Figure 3 of the manuscript. Then the red fluorescence of the VP was analysed from number of images taken from the triplicates of each sample group (cells only, cells with PLGA-VP and cells with PLGA-VP-TAT) using image J software. The VP fluorescence from each group is as shown in Fig. S3.

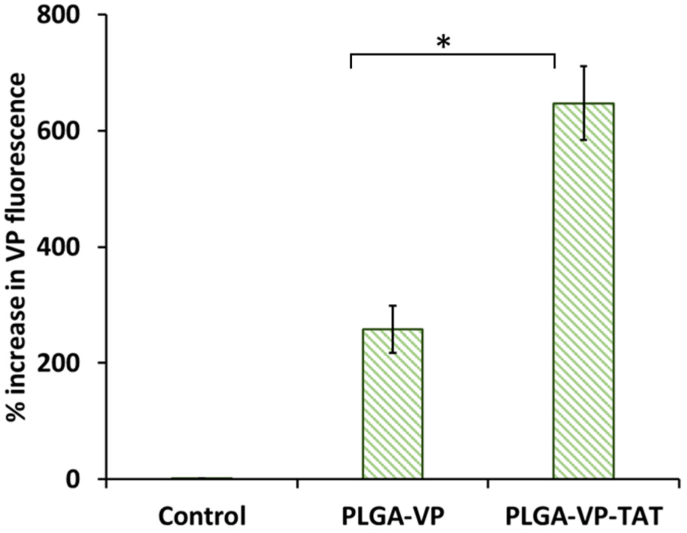

Figure S3. *Quantification of VP fluorescence inside the cells when incubated with PLGA-VP and PLGA-VP-TAT nanoparticles, *P<0.05.*

S4. MTS assay:

The *in vitro* dark cytotoxicity of the NPs was assessed using the CellTiter 96® AQueous One Solution MTS assay. The cells were grown to 80% confluence in 96-well plates and incubated in various NP formulations for 4 h. The MTS assay was read out by the 490 nm optical absorbance and the cell viability *versus* no-treatment controls was calculated [2]. The cell viability after RDT treatment was measured with the additional step where the cells were exposed to 4 Gy X-ray radiation immediately before the MTS assay. The cells were kept under minimum lighting conditions throughout to avoid phototoxicity.

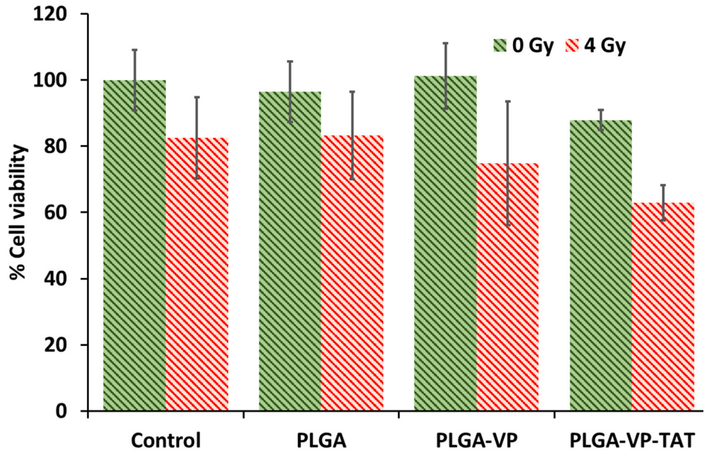

Figure S4. *In vitro dark cytotoxicity assessment, with and without X-ray exposure, normalized in each case to the no-treatment controls.*

S5. Live/ Dead assay:

The live/ dead assay is used to assess the cell death after RDT in various treatment groups using confocal microscopy. The green colour in the following images in Figure S5 indicates the live cells whereas the red colour indicates the dead cells.

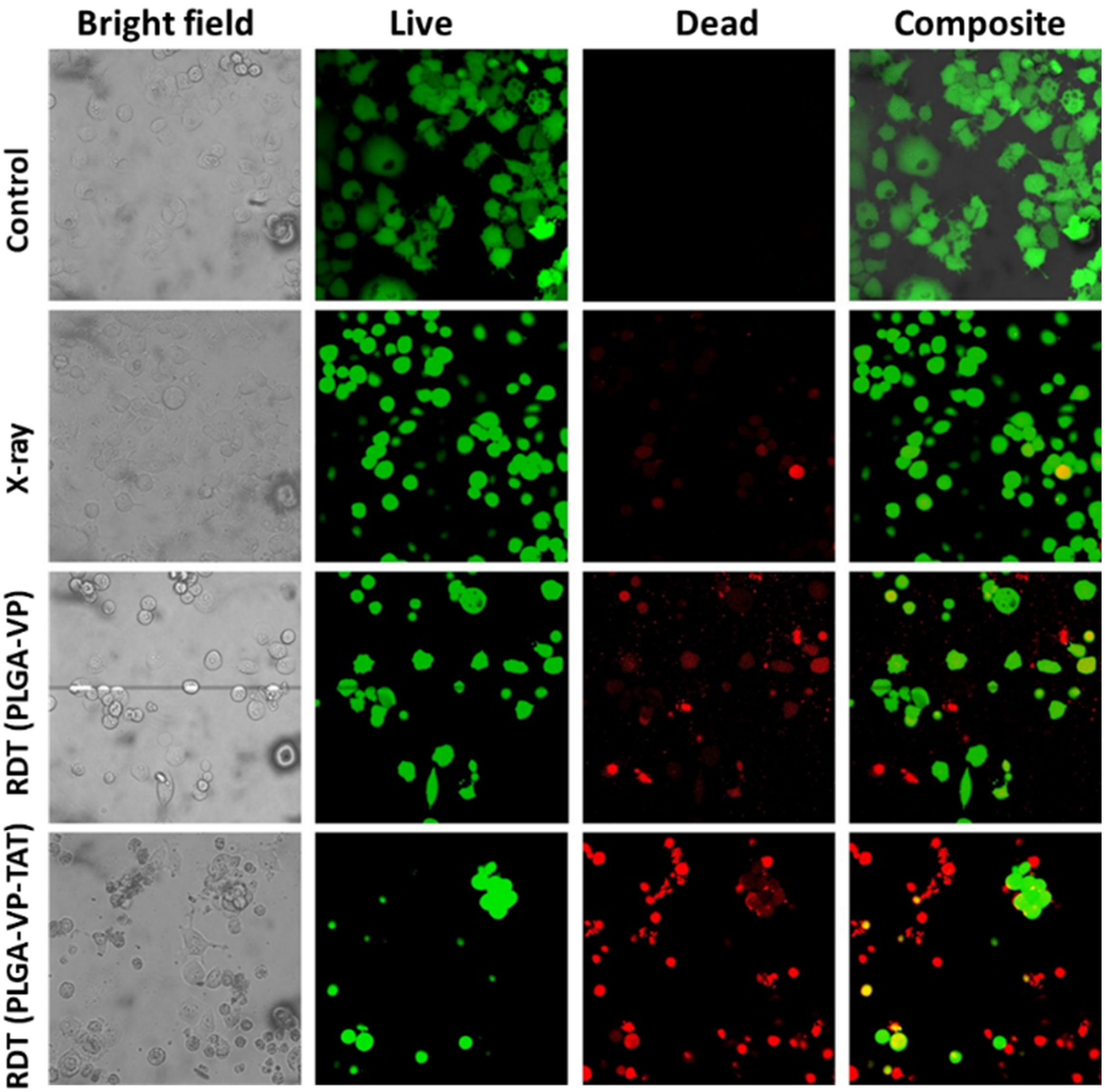

Figure S5. *Examples of confocal fluorescence micrographs to assess live and dead cells following various treatments. green: live, red: dead.*

S6. Singlet Oxygen Sensor Green (SOSG) assay:

Singlet oxygen Sensor Green (SOSG) probe is used to determine the singlet oxygen generation during RDT. The green colour in the Figure S6 indicates the SOSG fluorescence at 525 nm with 488 nm excitation. The blue colour indicates nucleus of the PANC-cells.

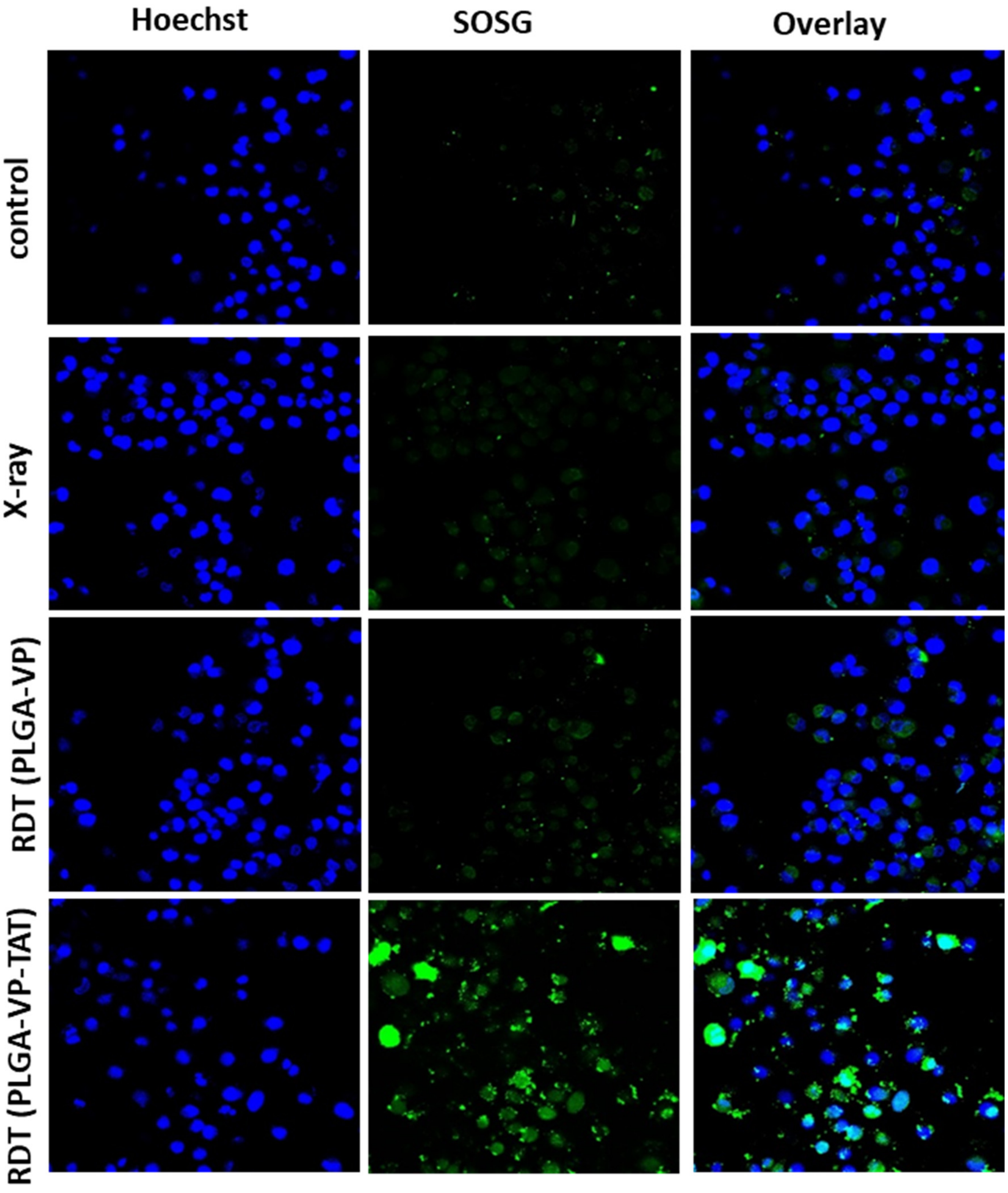

Figure S6. Examples of confocal fluorescence micrographs to assess singlet oxygen generation following various treatments. Blue: Nucleus, green: SOSG.

S7. Apoptosis/necrosis/live assay:

The Apoptotic/Necrotic/live assay provides a method for quantifying apoptotic (green), necrotic (red) cells and healthy (blue only) within the same cell population by confocal microscopy. Triple staining with Apopxin Deep Red Indicator (Ex/Em = 630/660 nm), Nuclear Green DCS1 (Ex/Em = 490/525 nm), and CytoCalcein Violet 450 (Ex/Em = 405/450 nm) was used to check the status of the cells. The RDT-treated cells and various controls were stained 1with the assay and imaged, as shown in Fig. S7.

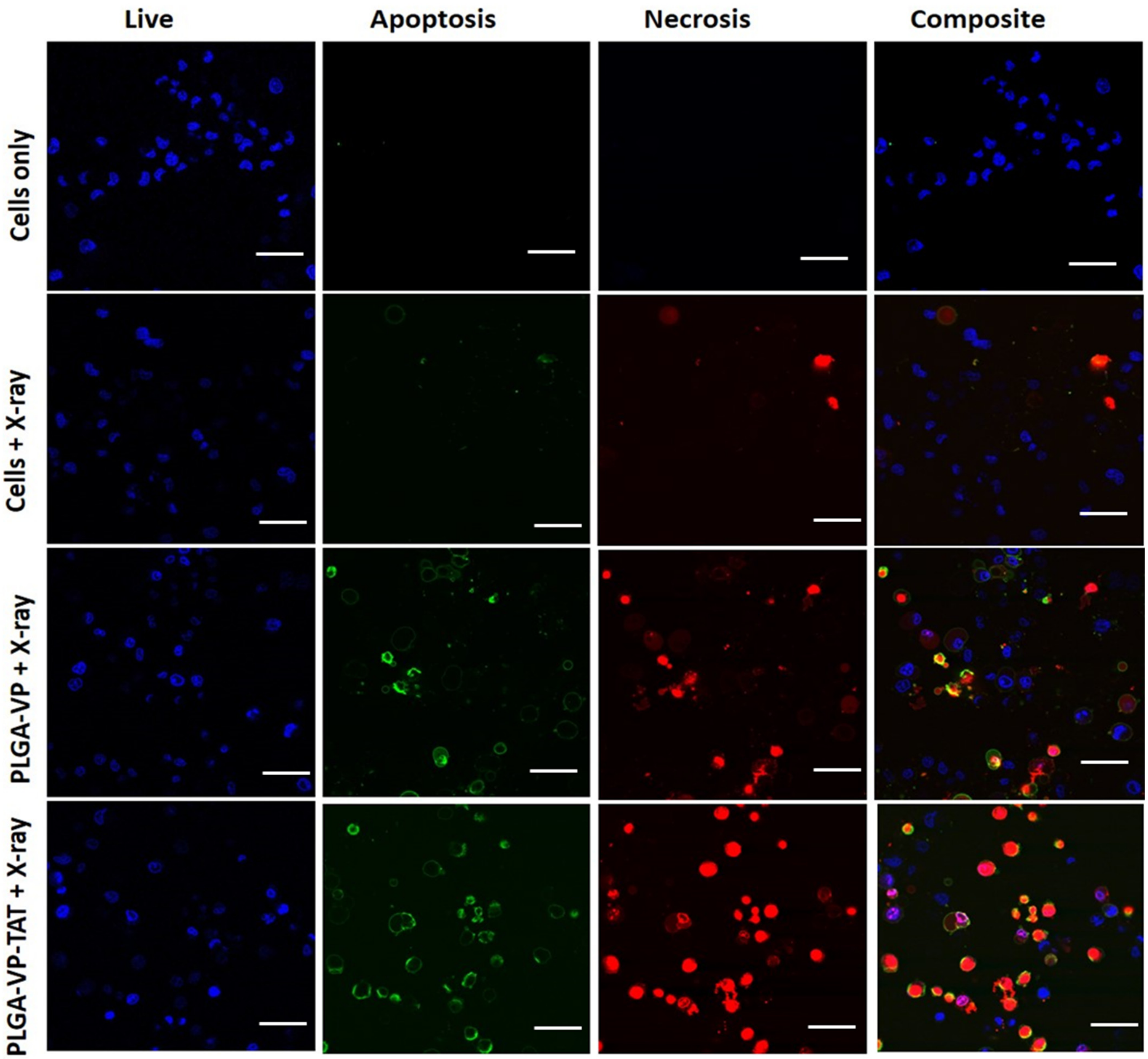

Figure S7. *Examples of confocal fluorescence micrographs to assess apoptotic, necrotic and live cells following various treatments. Blue: live, green: apoptotic, red: necrotic. Scale bar: 50 µm.*

S8: Lipid peroxidation:

A (BODIPY® 581/591 C11 reagent), the fluorescence emission of which changes from red to green upon oxidation, was used to assess lipid peroxidation following RDT, measuring the ratio of the fluorescence signal at 590 nm to that at 510 nm (Fig. S8). In the untreated controls, the high 590/510 ratio indicates a low basal level of lipid peroxidation. The ratio decreased after X-ray and RDT treatment, indicating significant oxidation of the dye and higher levels of lipid peroxidation.

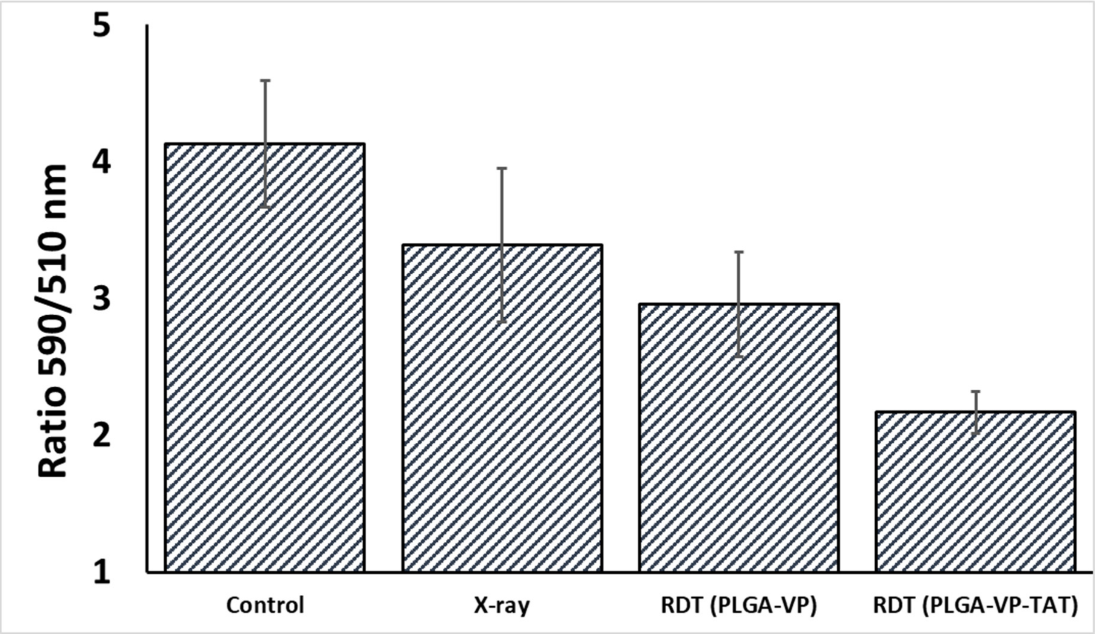

Figure S8. *The ratio of fluorescent intensity at 590 nm to that at 510 nm following treatment and in control untreated cells.*

References:

[1] R. Vankayala, C.L. Kuo, K. Nuthalapati, C.S. Chiang, K.C. Hwang, Nucleus-targeting gold nanoclusters for simultaneous in vivo fluorescence imaging, gene delivery, and NIR-light activated photodynamic therapy, Advanced Functional Materials, 25 (2015) 5934-5945.

[2] D. KARAKAŞ, F. Ari, E. Ulukaya, The MTT viability assay yields strikingly false-positive viabilities although the cells are killed by some plant extracts, Turkish Journal of Biology, 41 (2017) 919-925.
